# A pan-cancer characterization of immune-related NFIL3 identifies potential predictive biomarker

**DOI:** 10.7150/jca.88765

**Published:** 2024-01-12

**Authors:** Qin Fei, Xiaojun Zhang, Shutong Wang, Guang Shu, Gang Yin

**Affiliations:** 1 Department of Pathology, Xiangya Hospital, School of Basic Medical Sciences, Central South University, Changsha, China.; 2 Department of Xiangya School of Medicine, Central South University, Changsha, China.; 3 National Clinical Research Center for Geriatric Disorders, Xiangya Hospital, Central South University, Changsha, China.; 4 China-Africa Research Center of Infectious Diseases, School of Basic Medical Sciences, Central South University, Changsha, Hunan Province, China.

**Keywords:** NFIL3, prognosis, tumor microenvironment, immune infiltration, immunotherapy, pan-cancer analysis

## Abstract

**Background:** Nuclear factor interleukin 3 (NFIL3) mainly focuses on the regulation of the circadian rhythm and immune system. However, the potential role of NFIL3 in human cancers has not been studied extensively.

**Methods:** We retrieved original data from the TCGA, TARGET, and GTEx datasets via the UCSC Xena browser (http://genome.ucsc.edu/) and integrated them using R version 3.6.4. NFIL3 expression was assessed using resources such as UCSC, GEPIA (http://gepia.cancer-pku.cn/), Kaplan-Meier Plotter (KM Plotter; https://kmplot.com/), and the Human Protein Atlas (HPA; https://www.proteinatlas.org/) databases. To investigate the prognostic implications of NFIL3, we utilized GEPIA, Kaplan-Meier Plotter, and PrognoScan (http://www.abren.net/PrognoScan/) datasets. For a comprehensive analysis across multiple cancer types, we employed pan-cancer data from UCSC, examining associations between NFIL3 expression and genomic heterogeneity, tumor mutational burden (TMB), microsatellite instability (MSI), tumor purity, and neoantigens. Furthermore, we explored the relationships between NFIL3 expression and the infiltration of immune cells and the expression of immune checkpoint genes. In the context of ovarian cancer, we validated the expression and functional relevance of NFIL3. Cell Counting Kit 8 (CCK8) assays were conducted to assess cell proliferation, while scratch and transwell assays were employed to evaluate cell migration capabilities. We further examined the interaction between NFIL3 and the p53 signaling pathway through quantitative real-time polymerase chain reaction (qRT-PCR), Western blot analysis, immunofluorescence confocal, and Coimmunoprecipitation (Co-IP) assays.

**Results:** In general, NFIL3 expression in cancerous tissues exhibited diminished levels when compared to normal tissue samples. Notably, NFIL3 expression demonstrated a robust correlation with several pivotal aspects, including prognosis, immune cell infiltration, immune checkpoint-related genes, TMB, MSI, tumor purity, and the presence of neoantigens. Experimental investigations involving scratch assays, transwell assays, and assessments of cell proliferation in ovarian cancer cells have provided indications that NFIL3 may exert influence over cell migration and proliferation processes. Moreover, a substantial association between NFIL3 and the p53 signaling pathway was discerned through Kyoto Encyclopedia of Genes and Genomes (KEGG) analysis, with subsequent validation through qRT-PCR, Western blot analysis, immunofluorescence confocal, and co-immunoprecipitation (Co-IP) assays.

**Conclusions:** Therefore, we concluded NFIL3 may serve as a possible prognostic and immunological pan-cancer biomarker.

## Introduction

Cancer is poised to become the foremost global cause of mortality, representing a substantial impediment to the advancement of life expectancy in the 21st century^1^. The comprehension of the underlying mechanisms governing cancer initiation and progression has greatly diversified our arsenal of strategies for prevention and treatment. The intricate microenvironment within tumors, characterized by the presence of malignant cells, renders therapeutic interventions a formidable challenge. Given the ubiquity of tumors and the intricacies inherent in tumorigenesis, the identification of clinically precise targets remains paramount for a myriad of cancer types.

NFIL3 (Nuclear Factor, IL-3 Regulated; also known as E4BP4) is a protein encoded by the NFIL3 gene in humans. Originally identified as a protein binding to the adenovirus E4 promoter and the human IL3 gene promoter^2^, NFIL3 serves as a transcriptional regulator. It functions as a homodimer that binds to activating transcription factor (ATF) sites in numerous cellular and viral promoters. Additionally, NFIL3 represses the expression of PER1 and PER2 genes. Prior research^3^-^6^ has emphasized the critical roles of PER1 and PER2 in circadian rhythms, thereby implicating NFIL3 in the regulation of these biological processes.

Notably, heightened NFIL3 expression in cancer cell lines has been associated with a reduction in apoptosis occurrence and the inhibition of FOXO1 recruitment to specific genes linked to tumor suppression^7^. Furthermore, NFIL3 may hinder the recruitment of Proline Acid Rich (PAR) transcription factors to pro-apoptotic genes in colon cancer, as indicated by previous studies^8^.

While previous research^9^-^11^ has predominantly focused on its role in immune regulation, the connection between NFIL3 expression and its biological function in various human cancers, including ovarian cancer, has remained largely unexplored. Given the well-established link between immune function and the development and progression of tumors, especially in the context of anti-tumor immunity, this study aims to investigate the expression and clinical prognosis of NFIL3 and its correlation with the immune microenvironment across a spectrum of cancer types. This work clearly demonstrates the normal tissue with high NFIL3 expression compared to their cancer tissue counterparts in most carcinomas. And, NFIL3 is related to tumor prognosis and immune regulation. In conclusion, NFIL3 is capable of acting as a prognostic biomarker and is closely related to the immune system, which points to its potential as a cancer immunotherapy.

## Materials and methods

### Data Acquisition

All original data, including Pan-cancer RNA sequencing (RNA-seq), somatic mutation [Simple Nucleotide Variation (SNV), Copy Number Variation (CNV))], immune checkpoint, immune cell infiltration and tumor heterogeneity data were downloaded from the UCSC Xena (http://genome.ucsc.edu/) databases. All GEO data GEO series (GSE) studies were gathered from Gene Expression Omnibus (GEO; http://www.ncbi.nlm.nih.gov/geo/) database. All cancer type abbreviations of TCGA in Supplementary Table S1.

### NFIL3 mRNA expression analysis in different databases

NFIL3 gene expression between human cancers and non-paired normal tissue was obtained from the UCSC Genome Browser and GEPIA (http://gepia.cancer-pku.cn/) database. Meanwhile, human cancers and paired normal tissue were used to investigate the NFIL3 expression in Kaplan-Meier Plotter (KM Plotter; https://kmplot.com/). Additionally, we explored the relationship between NFIL3 expression and tumor pathology in GEPIA and a survival plot analysis of pan-cancer prognosis was performed using the "Survival Plots" module. The log2(TPM+1) for log scale algorithm was used in analysis in GEPIA.

Inclusion and exclusion criteria for our gene expression profiling study at UCSC were as follows: we obtained the TCGA TARGET GTEx pan-cancer dataset from the UCSC database (https://xenabrowser.net/), which includes 19,131 samples and 60,499 gene expressions. We specifically focused on expression data for the ENSG00000165030 (NFIL3) gene. Patient selection included samples from different tissue types and cancer categories, including normal solid tissue, primary solid tumors, primary tumors, normal tissue, primary blood-derived cancers-bone marrow, and primary blood-derived cancers-peripheral blood. We normalized the data using log2(x+0.001) transformation. To ensure data quality, we excluded cancer types with fewer than three samples in a given category. This meticulous sorting process resulted in a final dataset containing expression data for 34 different cancer types.

### The NFIL3 protein expression levels and protein-protein interactions

In order to determine NFIL3 protein expression level, the Human Protein Atlas (THPA, https://www.proteinatlas.org/) database was used to obtain immunohistochemistry (IHC) image. STRING (http://string-db.org/) database, which provides experimental and predicted protein-protein interaction (PPI) information, was used to explore the PPI networks, with a confidence cutoff of 0.4. In addition, the GeneMANIA (http://genemania.org/) database, which includes physical interactions, co-expression, prediction, co-localization, genetic interactions, pathways, and shared protein structural domains, was used for calculating the PPI interaction network. The connecting lines are drawn if the two genes have a relationship. The thickness of the lines represents the degree of similarity between two genes.

### Prognostic analysis of NFIL3

We utilized PrognoScan (http://www.abren.net/PrognoScan/), GEPIA, and Kaplan-Meier plotter database to conduct a Kaplan-Meier survival analysis. Patients were categorized as high or low expressors based on the median value of NFIL3 gene expression levels.

### Gene mutation and NFIL3 expression

Genetic variant characterization of NFIL3 was performed using dataset TCGA Pan-Cancer downloaded from UCSC. All somatic SNVs from GDC (https://portal.gdc.cancer.gov/) were carried out by the Mutect2 software. The copy number data from GDC were performed with the GISTIC software to identify the CNVs. The statistical significance of expression differences between tumors in SNV and CNVs was assessed using non-paired Wilcoxon Rank Sum and Signed Rank Tests, further tests were performed using kruskal.test among multiple groups of samples. Lollipop diagrams were generated using the maftools (version 2.2.10) R package to obtain protein domain information.

### NFIL3 expression with immune infiltration and immune checkpoints

The tumor immune cell infiltration score was determined with the xCell algorithm using data from TCGA Pan-Cancer downloaded from UCSC. Finally, 67 types of immune cell infiltration scores for 9555 tumor samples in a total of 39 types of cancer were included. We calculated the Pearson's correlation coefficient between NFIL3 expression and immune cell infiltration scores in individual tumors using the corr.test function of the R package psych (version 2.1.6) to identify significantly correlated immune infiltration scores. The combined cohort of TCGA, TARGET, and GTEx samples were obtained from UCSC Xena browser were used to investigate the relationship between NFIL3 expression and immune checkpoint genes. By searching published article^12^, 24 inhibitory and 36 stimulatory markers of the immune systems were selected to construct association networks of NFIL3 expression and immune checkpoint genes. A Pearson's correlation coefficient analysis heat map of the NFIL3 gene expression with immune infiltration scores and immune checkpoint-related genes in multiple cancers was generated.

### Pan-cancer analysis of the relationship between the NFIL3 gene expression and tumor heterogeneity

The TMB, MSI, purity and neoantigen data were obtained from TCGA Pan-Cancer downloaded from UCSC. Correlation analysis between the NFIL3 expression and TMB, MSI, purity and neoantigen was performed using pearson's method.

### Pathway analysis

Enrichment analysis for KEGG was performed with the GeneCodis tool (http://genecodis.dacya.ucm.es/). The intersection of potential targets of NFIL3 and the differential gene expression between normal ovary and ovarian cancer tissue were used as input. The top 10 enriched pathways in the gene sets were displayed. The target genes of NFIL3 were acquired from the hTFtarget database (http://bioinfo.life.hust.edu.cn/hTFtarget).

### Cell lines and culture

Human OV cell lines (OVCAR3, MR182, TR182, A2780, SKOV3, HO 8910 and HO 8910PM) and jurkat cells (T cell line) were maintained by the laboratory of Professor Gang Yin (Changsha, China) and cultured in RPMI-1640 (Basal Media, Shanghai, China) and 293FT were cultured in DMEM medium (Basal Media, Shanghai, China). MR182 (Type II Mature Epithelial Ovarian Cancer cell) was given by Professor Gil Mor, who works in The C.S. Mott Center for Human Growth and Development and President-American Society for Reproductive Immunology. The media were supplemented with 10% fetal bovine serum (FBS) (Gibco, Carlsbad, CA). At 37 degrees Celsius, all cells were cultured in a humidified 5% CO2 incubator.

### RNA extraction and quantitative real‐time PCR analysis

RNA was extracted using a total RNA extraction reagent (Vazyme Biotech). Reverse transcription was performed with a GoScript Reverse Transcription System (Promega, Madison, WI, USA). Real-time qPCR was performed with qPCR Master Mix (Vazyme Biotech) in an Applied Biosystems 7500 Real-Time PCR System. The primers used were designed by Sangon Biotech (Shanghai, China), NFIL3, forward: 5'-TGGAGAAGACGAGCAACAGGTC-3' and reverse: 5'-CTTGTGTGGCAAGGCAGAGGAA-3'; p53, forward: 5'-CCTCAGCATCTTATCCGAG TGG-3' and reverse: 5'-TGGATGGTGGTACAGTCAGAGC-3'; p21, forward: 5'-AGGTGG ACCTGGAGACTCTCAG-3' and reverse: 5'-TCCTCTTGGAGAAGATCAGCCG-3'; Bax, forward: 5'- TCAGGATGCGTCCACCAAGAAG-3' and reverse: 5'-TGTGTCCACGGCGG CAATCATC-3' and GAPDH, forward: 5'-GTCTCCTCTGACTTCAACAGCG-3' and reverse: 5'-ACCACCCTGTTGCTGTAGCCAA-3'.

### NFIL3 CRISPR/Cas9 knockout plasmid

Candidate single-guide RNAs (sgRNAs) targeting the second exons of NFIL3 were selected using CRISPick database (https://portals.broadinstitute.org/gppx/crispick/public). The sgRNA sequence was synthesized and annealed, then ligated into lenti-CRISPR V2, which had been linearized by BsmBI. Thermocycler settings were as follows: 37 °C for 30 min, 95 °C for 5 min, then ramping down to 25 °C at 5 °C /min. The plasmids were transformed into E. coli DH5a, and the sequences were confirmed by Sanger sequencing by Qingke Biotechnology Company (Changsha, China). The primers used are shown in Supplementary Table 2.

### Overexpression plasmid cloning

The following expression plasmids were generated: pEGFP-N1-NFIL3, *pmCherry*-*C1*-P53. The primers used in construction plasmids were designed by Sangon Biotech (Shanghai, China), *pmCherry-C1*-P53, forward: ATTCTGCAGTCGACatggaggagccgcagt and reverse: GATCCCGGGCCCGCGtcagtctgagtcaggcccttctg, pEGFP-N1-NFIL3, forward: ATTCTGCAGTCGACGGTACCatgcagctgagaaaaatgcagacc and reverse: GATCCCGGGCCCGCGGTAC gtacccagagtctgaagcagagattgg.

### CCK8 assay

According to the manufacturer's instructions, we performed the Cell Counting Kit 8 (CCK8) experiment for *in vitro* proliferation assays as previously^13^**.** CCK8 Kit was purchased from Shanghai Seven Sea Biotechnology Co., Ltd.

### Transwell assay

Transwell assay was performed using Corning Transwell Assay Kit. For the assay of NFIL3-regulated tumor cell migration ability, HO 8910 and HO 8910PM cells were collected 24 h after transfection into the upper chamber of a 24-well plate containing 200 μL of FBS-free medium (3 × 10^5^ cells/well). For the assay of NFIL3 ability to attract jurkat cells, A2780 or SKOV3 cells overexpressing or knocking down NFIL3 were seeded into the lower chamber. 24 h after transfection, Jurkat cells were introduced into the upper chamber of a 24-well transwell plate at 5 × 10^6^ cells/mL for NFIL3 overexpression and 1 × 10^7^ cells/mL for NFIL3 knockdown, all in 200 μL of medium. Both assays were supplemented with 800 μL of medium containing 20% FBS in the lower chamber. After 24 hours of incubation, non-migrating cells were removed with a cotton swab. The remaining cells were subsequently fixed with 4% formaldehyde and stained with crystal violet. Cell migration ability was subsequently assessed and quantified using the analysis tool in Adobe Photoshop 2020.

### Wound-scratch assay

Briefly, 6×10^5^ ovarian cancer cells were inoculated into 6-well plates and cultured until cell confluence was >95%. The cell layer was scratched at the bottom of the wells with a sterile 10 µl pipette tip, rinsed three times with PBS, and serum-free medium was added to the chamber of the plate, and the cells were treated with different time points. The migration of cells towards the center of the scratch was observed under a phase contrast microscope (magnification, ×40). Quantification was performed using the analysis tool in Adobe Photoshop 2020.

### Western blot

Western blot was performed as described previously^13^. Cells were cultured in 60 mm dishes to approximately 80% confluence and then lysed with ice-cold lysis RIPA buffer (P0013B) containing 1% protease inhibitor cocktail and the concentration of the supernatant was determined by BCA. After loading the target proteins onto an SDS poly-acrylamide gel, the proteins were transferred from the gel to a PVDF membrane. Subsequently, the membranes were incubated at 4 °C overnight with NFIL3 (11773-1-AP, Wuhan Sanying Biotechnology), p53 (cat. no. sc-126), p21 (cat. no. Sc-6246), Bax (cat. no. sc-7480), GFP antibody (Utibody, UM3002) and GAPDH (Utibody, UM4002) after being blocked with 5% BSA for 2 h. After washed 3 times with TBST, the membranes were incubated with secondary antibody (Zhengneng Biotechnology, cat. no. 8F10) for 90 min at room temperature. Protein bands were detected and analyzed using enhanced ECL chemiluminescence. Digital images were taken by a MiniChemi from SAGECREATION, Beijing, China.

### Co-immunoprecipitation (Co-IP)

We conducted the Co-IP using protein A/G PLUS Agarose beads (Santa Cruz Biotechnology, SC-2003). Briefly, samples were extracted from SKOV3 (10 cm dishes per sample) using IP lysis buffer. Samples were incubated with mouse GFP antibody (1:100, Utibody, UM3002) or mouse isotype control IgG (1:200, Abclonal, AC005, China) overnight at 4 °C with gentle rotation. Add 40 μl of protein A/G beads to the samples and incubate at 4 °C overnight. The beads were washed five times with cold IP lysis buffer. The eluted proteins were then used for Western blotting.

### Immunofluorescence analysis

Immunofluorescence analysis experiments were performed with cells co-transfected with pmCherry-C1-P53 plasmids and pEGFP-N1-NFIL3 for 48h. Fixed cells were washed with 1×PBS for 3 × 5 minutes. An analysis was performed on immuno-fluorescent images of DAPI (Solaibao Biological Technology Co., Ltd.) stained nuclei. Confocal fluorescence images were randomly obtained with a confocal microscope.

### Immunohistochemistry

Immunohistochemistry was performed as previously described 13. Ovarian tissue sections were deparaffinized, hydrated, quenched and blocked with 1% normal goat serum, and then incubated with NFIL3 antibody (1:150) at 4℃ overnight. Immunohistochemical staining was performed using the SP-9001 kit (Zhongshan Jinqiao, Beijing, China). NFIL3 staining was evaluated in five fields of view under high magnification (200×). The intensity of staining was categorized as 0 (negative), 1 (weak), 2 (moderate) or 3 (strong); the percentage of stained cells was categorized as 1 (0-25%), 2 (26-50%), 3 (51-75%) or 4 (> 75%), and the composite score was calculated by the percentage of staining × the intensity of staining.

### Statistical analysis

Each experiment was repeated at least three times, independently. Quantitative data are presented as mean±SEM. Differences between two groups were compared by Student's t -test. Gene mRNA expression in bioinformatics analysis was examined using Wilcoxon Rank Sum and Signed Rank Tests in non-paired samples. The analysis was carried out with R software (version 3.6.4) and GraphPad Prism 7. The remaining statistical methods for each figure are given in the respective section of the materials and methods or the figure legends. We considered a difference of *p* < 0.05 to be statistically significant.

## Results

### Analysis of NFIL3 expression in Pan-Cancer

**Figure 1:**
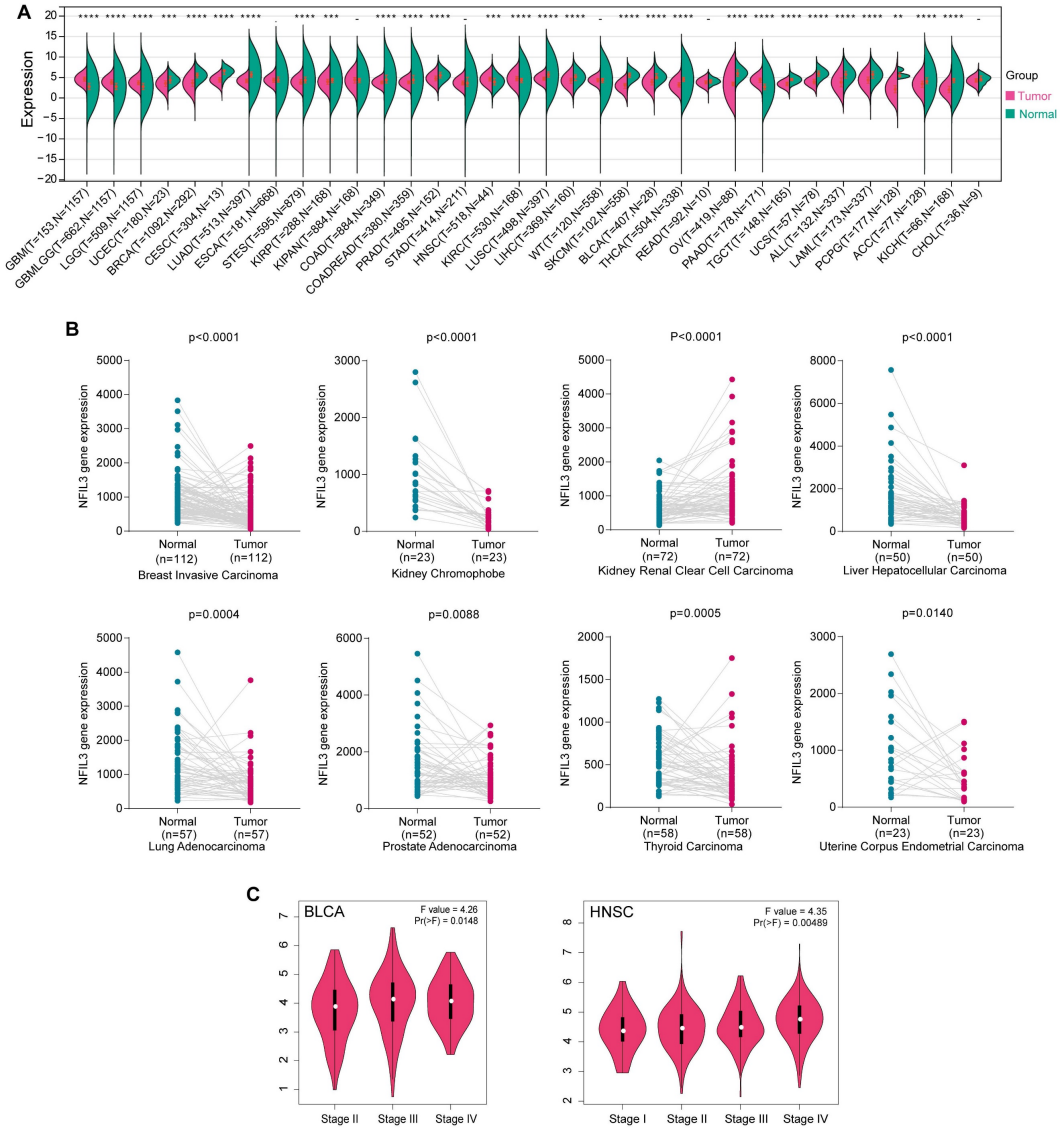
** NFIL3 expression levels in various cancer and different pathological stages. (A)** UCSC data on NFIL3 expression in different cancer types from TCGA, Target and GTEx database. **(B)** NFIL3 mRNA expression in paired cancer tissue and normal tissue from KM-plotter. **(C)** Using TCGA data, the expression levels of the NFIL3 gene were analyzed according to the major pathological stages (stage I, stage II, stage III, and stage IV) of BLCA and HNSC in GEPIA. Log2 (TPM+1) was applied for log-scale. (*p< 0.05, **p < 0.01, ***p < 0.001).

Limited research has been conducted to investigate the expression of NFIL3 across diverse cancer types. In an effort to provide a more comprehensive assessment of NFIL3 expression in cancer, we conducted a comparative analysis of NFIL3 mRNA expression across 34 cancer types, utilizing datasets from TCGA, TARGET, and GTEx, accessible through the UCSC Xena Browser. As illustrated in Figure 1A, NFIL3 mRNA expression exhibited an elevation in GBM, GBMLGG, LGG, HNSC, KIRC, and PAAD. In contrast, NFIL3 mRNA expression displayed diminished levels in 22 other tumors, including but not limited to UCEC, BRCA, CESC, LUAD, STES, KIRP, COAD, and COADREAD.

To assess NFIL3 expression in matched cancer and adjacent normal tissues, we conducted a comparative analysis of NFIL3 mRNA expression using the Kaplan-Meier Plotter database. The results revealed significant differences in NFIL3 mRNA expression in paired samples, specifically in BRCA (*p* < 0.0001), KICH (*p* < 0.0001), KIRC (*p* < 0.0001), LIHC (*p* < 0.0001), LUAD (*p* = 0.0004), PRAD (*p* = 0.0088), THCA (*p* = 0.0005), and UCEC (*p* = 0.0140) (Figure 1B). Notably, an opposing trend was observed exclusively in THCA, while the remaining outcomes were largely consistent with the analyses conducted using data from TCGA, TARGET, and GTEx in the UCSC Xena Browser.

In addition, NFIL3 expression was evaluated in different cancer stages, and the NFIL3 expression level was significantly higher in the advanced stages of BLCA (*p*=0.0148) and HNSC (*p*=0.00489) (Figure 1C).

### An analysis of NFIL3 protein levels

**Figure 2:**
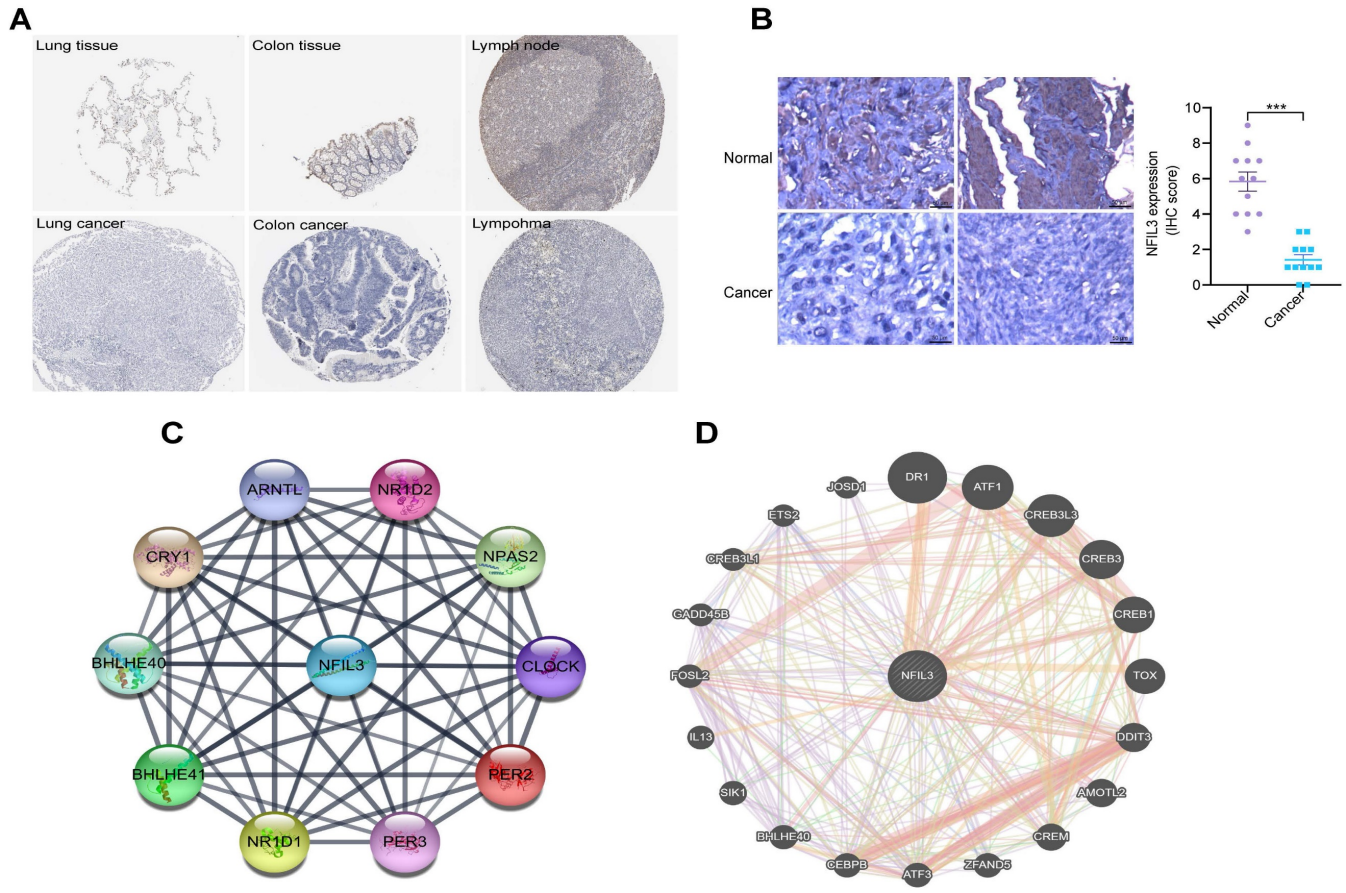
** Immunohistochemical analysis of NFIL3 protein expression and PPI network construction. (A)** Immunohistochemical stains slices in human cancers and corresponding normal tissues from the HPA database. Top row: normal lung, colon, and lymph node; bottom row are corresponding cancer tissues. **(B)** Immunohistochemical staining for NFIL3 in ovarian control and cancer. ***p < 0.001. **(C)** Using the STRING online database, 10 genes were selected and used to construct the PPI network. A confidence score > 0.4 was set as significant. PPI, protein-protein interaction. **(D)** PPI network of the related genes of NFIL3 was constructed by GeneMANIA.

Utilizing the HPA database, we conducted an examination of NFIL3 protein levels in various tumors, revealing a decrease in NFIL3 levels in lung, colon cancers and lymphoma (Figure 2A). Additionally, we assessed NFIL3 protein expression using paraffin sections from 12 ovarian normal controls and 12 ovarian cancer samples (Figure 2B), and the immunohistochemical scores were assessed by three pathologists. A protein-protein interaction (PPI) network was subsequently constructed in the STRING database, illuminating NFIL3's associations with NR1D1, NR1D2, PER3, NPAS2, CRY1, BHLHE40, PER2, ARNTL and BHLHE41 proteins (Figure 2C). Furthermore, correlations were evaluated using the GeneMANIA database, revealing NFIL3's co-expression with DR1, ATF1, CREB3L3, CREB3, CREB1, TOX, DDIT3, AMOTL2, CREM, ZFAND5, ATF3, CEBPB, BHLHE40, SIK1, IL13, FOSL2, GADD45B, CREB3L1, ETS2 and JOSD1 (Figure 2D).

### A multifaceted prognostic value for NFIL3

**Figure 3:**
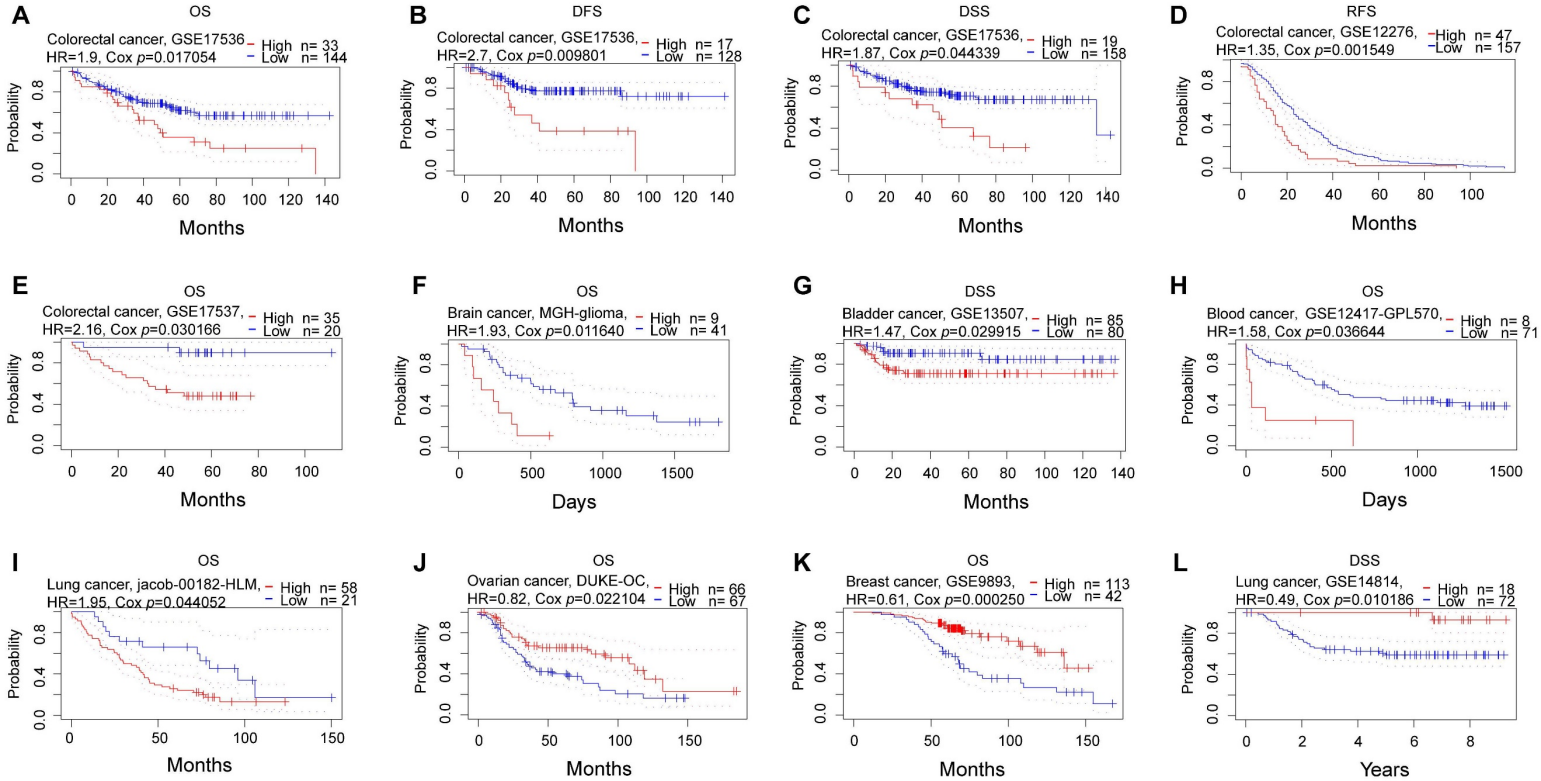
** Prognostic analysis of NFIL3 in various cancers using the PrognoScan database. (A-C)** Correlation between NFIL3 expression and OS, DFS and DSS in colorectal cancer (n = 177) based on GSE17536 dataset. **(D-E)** Relationship between NFIL3 expression and RFS in GSE12276 cohorts (n=204) and OS in GSE17537 cohorts (n=55) in colorectal cancer. **(F)** Correlation between NFIL3 expression level and OS in brain cancer, specifically MGH-glioma. **(G-I)** Elevated NFIL3 expression was linked to poorer survival outcomes, including DSS in bladder cancer, OS in blood cancer, and OS in lung cancer. **(J)** The association between NFIL3 expression and OS in Ovarian cancer (n = 133). **(K)** The association between NFIL3 expression and OS in Breast cancer (n = 155). **(L)** High NFIL3 expression indicated better survival in terms of DSS in lung cancer (n = 90).

Subsequently, we conducted a comprehensive analysis of the prognostic significance of NFIL3 across various cancers in pan-cancer datasets. In this endeavor, we explored the association between NFIL3 expression and prognosis using the PrognoScan database. The results unveiled statistically significant differences in breast cancer, colorectal cancer, ovarian cancer, brain cancer, bladder cancer, blood cancer, and lung cancer. Particularly in colorectal cancer, NFIL3 expression exhibited a correlation with poorer prognosis, as evidenced by significant outcomes in overall survival (OS; total number = 177, HR = 1.9, Cox *P* = 0.017054), disease-free survival (DFS; total number = 145, HR = 2.7, Cox *P* = 0.009801), and disease-specific survival (DSS; total number = 177, HR = 1.87, Cox *P* = 0.044339) in GSE17536, as well as relapse-free survival (RFS; total number = 204, HR = 1.35, Cox *P* = 0.001549) in GSE12276 and OS (total number = 55, HR = 2.16, Cox *P* = 0.030166) in GSE17537 (Figure 3A-3E).

Consistently, NFIL3 expression was correlated with a poor prognosis in brain cancer (MGH-glioma) (OS: total number = 50, HR = 1.93, Cox *P* = 0.011640) (Figure 3F), bladder cancer (DSS: total number = 165, HR = 1.47, Cox *P* = 0.029915) (Figure 3G), blood cancer (OS: total number = 79, HR = 1.58, Cox *P* = 0.036644) (Figure 3H) and OS in lung cancer (OS: total number = 79, HR = 1.95, Cox *P* = 0.044052) (Figure 3I). Conversely, NFIL3 had a protective role in the other 3 cancer types, including ovarian cancer (OS: total number = 133, HR = 0.82, Cox P = 0.022104) (Figure 3J), breast cancer (OS: total number = 155, HR = 0.61, Cox P = 0.000250) ((Figure 3K) and DSS in lung cancer (DSS: total number = 90, HR = 0.49, Cox P = 0.010186) (Figure 3L).

### Analysis of NFIL3 gene mutation and expression level

**Figure 4:**
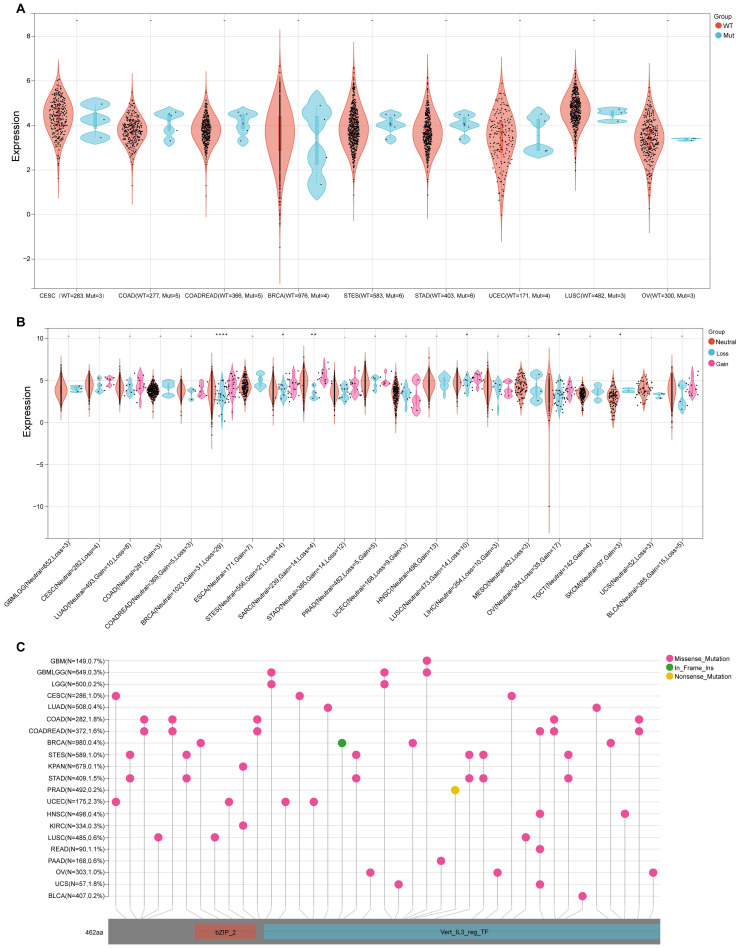
** Genetic alterations of NFIL3 in pan-cancer. (A)** Violin plot showing the differences in NFIL3 expression between NFIL3 mutation and wild-type groups. Orange distributions: wild-type (WT); light blue distributions: Mutation (Mut). The vertical axis is the gene expression level for NFIL3 and the horizontal axis is the NFIL3 mutation status and number of patients. The violin shape is the frequency distribution of gene expression levels: the inside boxplot represents the median (short transverse line), interquartile range (the box edge) and 95% confidence interval (the solid line). **(B)** Violin plot showing the gain-loss mutations in NFIL3 expression across 21 kinds of cancers. Orange distributions: neutral; light blue distributions: Loss; Pink distributions: Gain. *p < 0.05, **p < 0.01, ***p < 0.001. **(C)** Distribution of somatic mutations of NFIL3 genes. The schematic represents domain information and mutation positions are marked by "lollipops". Red lollipops represent Missense mutations while green lollipops represent In Frame Ins and yellow lollipops represent Nonsense mutations.

As SNV status was often associated with abnormal gene expression^14^, we first checked the SNV status of NFIL3. We conducted an in-depth study using the TCGA Pan-Cancer data downloaded from UCSC and found that NFIL3 mutated in 9 types of cancers, including CESC (WT = 283, Mut = 3), COAD (WT = 277, Mut = 5), COADREAD (WT = 366, Mut = 5), BRCA (WT = 976, Mut = 4), STES (WT = 583, Mut = 6), STAD (WT = 403, Mut = 6), UCEC (WT = 171, Mut = 4), LUSC (WT = 482, Mut = 3), OV (WT = 300, Mut = 3) (Figure 4A). Furthermore, CNV analysis of gene gain and loss in samples in various cancers were analyzed to examine NFIL3 mutation. The analysis revealed that considerable differences in CNV mutation were observed among 6 types of cancers, including GBMLGG (Neutral = 652, Loss = 3), CESC (Neutral = 282, Loss = 4), LUAD (Neutral = 493, Loss = 8), COAD (Neutral = 281, Gain = 3), COADREAD (Neutral = 369, Gain = 5, Loss = 3), BRCA (Neutral = 1023, Gain = 31, Loss = 29), ESCA (Neutral = 171, Gain = 7), STES (Neutral = 556, Gain = 21, Loss = 14), SARC (Neutral = 239, Gain = 14, Loss = 4), STAD (Neutral = 385, Gain = 14, Loss = 12), PRAD (Neutral = 482, Gain = 5, Loss = 5), UCEC (Neutral = 168, Loss = 9, Gain = 3), HNSC (Neutral = 498, Gain = 13), LUSC (Neutral = 473, Gain = 14, Loss = 10), LIHC (Neutral = 354, Loss = 10, Gain = 3), MESO (Neutral = 82, Loss = 3), OV (Neutral = 364, Loss = 35, Gain = 17), TGCT (Neutral = 142, Gain = 4), SKCM (Neutral = 97, Gain = 3), UCS (Neutral = 52, Loss= 3), BLCA ((Neutral = 385, Gain = 15, Loss = 5) (Figure 4B). To better understand the mutational map of NFIL3 in different cancer types across protein domains, lollipop plots were plotted to find mutations located between 0 and 462 amino acids. The results showed that missense mutation present in GBM, GBMLGG, LGG, CESC, LUAD, COAD, COADREAD, BRCA, STES, KPAN, STAD, UCEC, HNSC, KIRC, LUSC, READ, PAAD, OV, UCS and BLCA, in frame insertion (In_Frame_Ins) mutation presented in BRCA, nonsense mutation presented in PRAD (Figure 4C). WT = wild type, Mut = mutant

### Correlation of immune cell infiltration and immune checkpoint-related genes with NFIL3 expression

**Figure 5:**
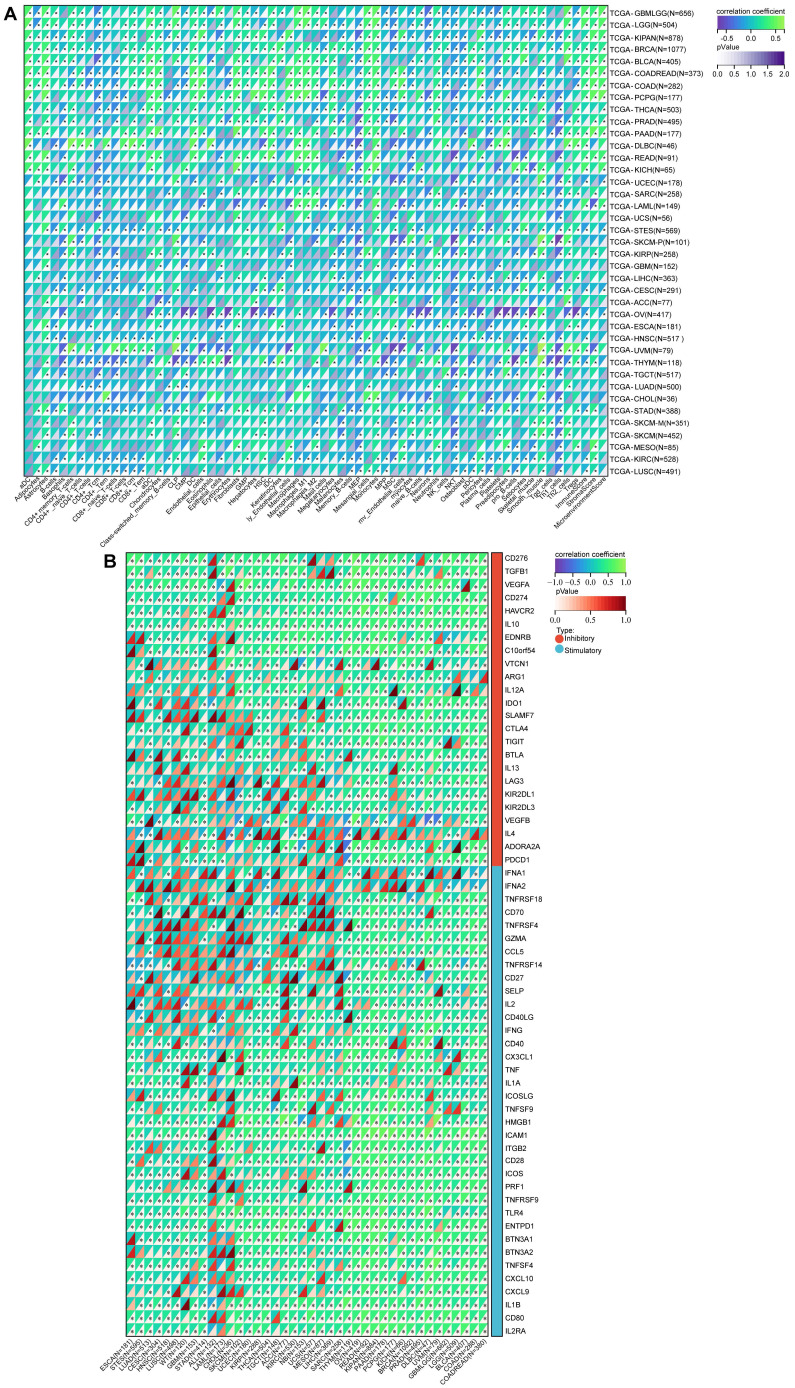
** A pan-analysis of immune cells infiltration and immune checkpoints. (A)** The coorelation of various immune cells infiltration heatmap and NFIL3 expression in tumors. **(B)** Heatmap showing correlation between NFIL3 expression and 60 types of immune checkpoint genes in TCGA tumors. *p < 0.05, **p < 0.01, ***p < 0.001.

Previous research has underscored the pivotal roles played by NFIL3 as regulators of both inflammatory responses^15^, ^16^ and circadian clock mechanisms^17^. The intricate interplay between immune cells and tumor cells has been long acknowledged^18^. Armed with this foundational knowledge, we embarked on a comprehensive pan-cancer analysis to explore the intricate relationship between NFIL3 expression and immune cell infiltration levels, drawing upon the TCGA Pan-Cancer datasets. Employing the xCell algorithm, we estimated infiltration scores for a total of 67 distinct immune cell types. Our findings unveiled a significant correlation between NFIL3 expression and infiltrating immune cells in 39 cancer types, encompassing GBM, GBMLGG, LGG, CESC, LUAD, COAD, COADREAD, LAML, BRCA, ESCA, and others. Moreover, our results indicated that NFIL3 expression exhibited significant correlations with ImmuneScores in 23 cancer types, MicroenvironmentScores in 18 cancer types, and StromaScores in 19 cancer types (Figure 5A).

To estimate the correlation between NFIL3 expression and the tumor microenvironment (TME) in pan-cancer, we investigated the association between NFIL3 expression and two major types (inhibitory and stimulatory) of immune regulators^12^. Our findings displayed that NFIL3 was mostly relevant to immune checkpoints in the majority of cancers, including ESCA, STES, LUAD, CESC, HNSC, LUSC, WT, GBM, STAD, ALL, etc. (Figure 5B).

### Expression of NFIL3 is associated with tumor mutation burden (TMB) and microsatellite instability (MSI), tumor purity and neoantigen

Tumor mutation burden (TMB) and microsatellite instability (MSI) are pivotal determinants in the initiation and progression of tumors. Within tumor microenvironments, TMB, MSI, tumor purity, and neoantigens are intricately linked to anti-tumor immune responses and may serve as predictive markers for the efficacy of tumor immunotherapy^19^-^21^. Therefore, we conducted an examination of the correlation between TMB, MSI, tumor purity, neoantigens, and NFIL3 expression.

**Figure 6:**
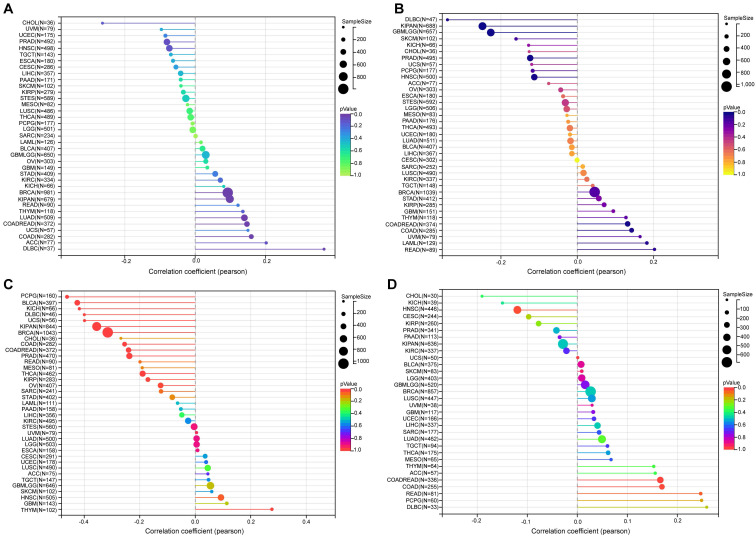
** The relationship between NFIL3 expression and tumor heterogeneity in TCGA Pan-cancer.** The relationship between NFIL3 expression and TMB **(A)**, MSI **(B)**, purity **(C)** and neoantigen **(D)** in various cancer types. TMB, tumor mutational burden; MSI, microsatellite instability. *p<0.05, **p<0.01, ***p<0.001.

Our findings revealed a significant positive correlation between NFIL3 expression and TMB in various cancers, including LUAD, COAD, COADREAD, BRCA, KIPAN, and DLBC (Figure 6A). Microsatellite instability (MSI) arises from the insertion or deletion of repeat units at specific microsatellite loci within tumor tissues due to functional deficiencies in DNA mismatch repair. MSI, often associated with DNA mismatch repair deficiencies, is a crucial clinical marker in oncology. Our analysis unveiled significant positive correlations between NFIL3 expression and MSI in specific cancers such as COAD, COADREAD, and LAML. Conversely, an inverse correlation was observed in GBMLGG, KIPAN, PRAD, HNSC, and DLBC (Figure 6B).

In addition to tumor cells, tumor tissues encompass non-tumor elements like immune cells, stromal cells, and interstitial cells, collectively impacting tumor initiation and progression. Tumor purity, reflecting the proportion of tumor cells within a sample, significantly influences clinical characteristics, genomic expression, and the biological features of tumor patients. Therefore, it is imperative to consider the influence of tumor purity in sample analysis. Our analysis of NFIL3 expression and tumor purity indicated significant positive correlations in HNSC and THYM. Conversely, we observed significant inverse correlations between NFIL3 expression and tumor purity in COAD, COADREAD, BRCA, KIRP, KIPAN, PRAD, THCA, OV, PCPG, UCS, BLCA, KICH, and DLBC (Figure 6C). Neoantigens, derived from non-synonymous mutations, are tumor-specific antigens with high immunogenicity and substantial tumor heterogeneity. They represent attractive targets for tumor immunotherapy, with neoantigen vaccines undergoing clinical trials for various solid tumors. Our exploration of the association between NFIL3 expression and neoantigens revealed strong positive correlations in COAD, COADREAD, and READ, while negative correlations were observed in HNSC (Figure 6D).

### NFIL3 functional validation in ovarian cancer cell lines

The selection of ovarian cancer as a model for studying NFIL3 was driven by several compelling factors. Firstly, previous reports have established the regulatory role of the circadian clock in ovarian cancer^22^, and NFIL3's involvement in circadian rhythm regulation is well-documented. Secondly, the specific role of NFIL3 in ovarian cancer had not been previously investigated. Lastly, there were significant differences in NFIL3 expression and prognosis between cancer and corresponding normal tissues in a substantial portion of cancer types.

**Figure 7:**
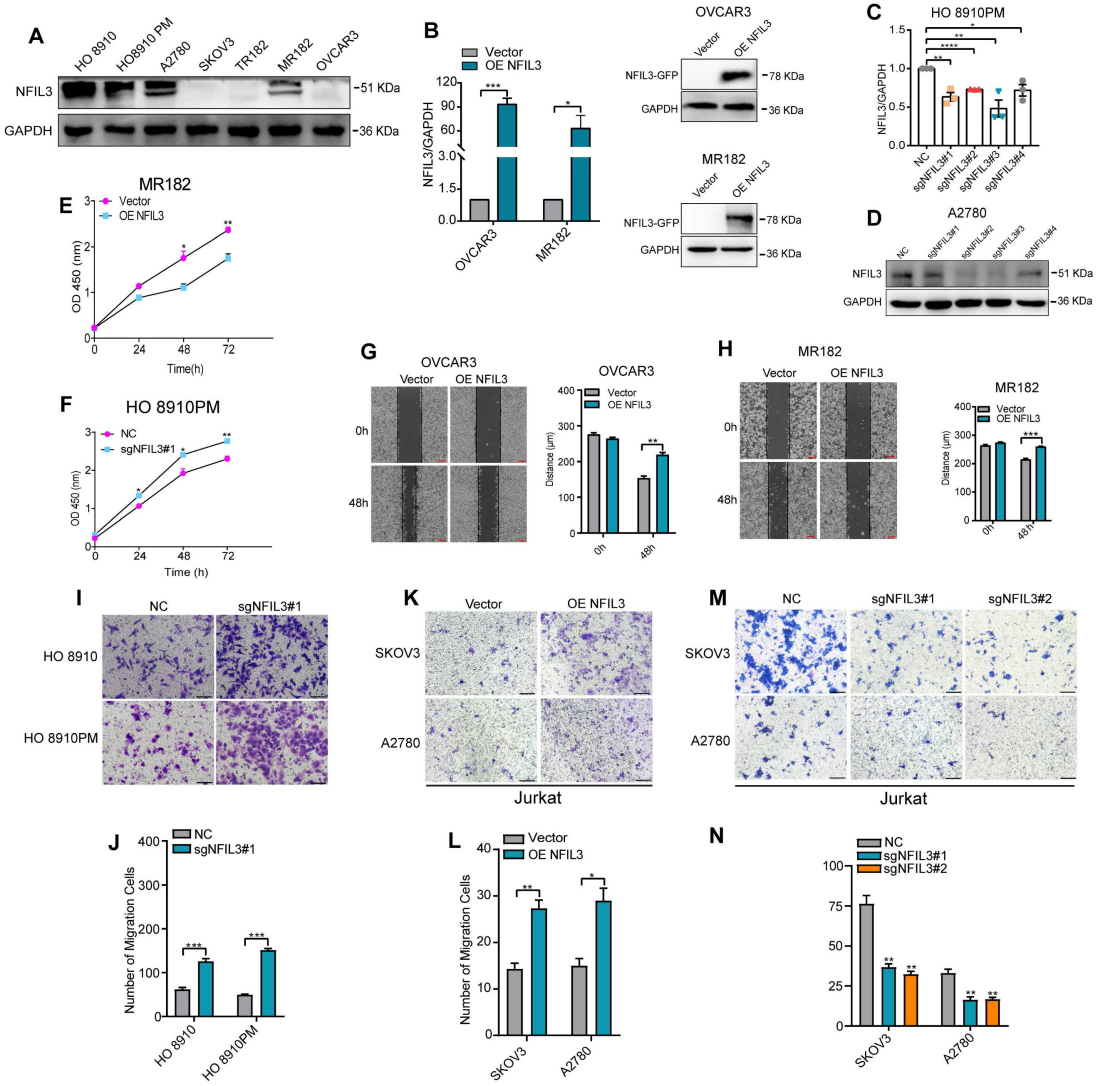
** Effect of NFIL3 on the biological behavior of ovarian cancer cell and the migratory ability of Jurkat cells.** The cell proliferation activity and migratory ability assay by overexpression or knockdown NFIL3 gene in ovarian cancer cell lines. **(A)** The expression of NFIL3 protein in human ovarian cancer cell lines was analyzed by western blot. **(B)** Verification of NFIL3 overexpression in OVCAR3 and MR182 cells using qRT-PCR analysis (left panel) and western blot (right panel). **(C)** Verification of NFIL3 mRNA knockdown effect in HO 8910PM cells using qRT-PCR analysis. **(D)** Verification of NFIL3 protein knockdown effect in A2780 cells using western blot analysis. **(E-F)** Cell proliferation assay in MR182 and HO 8910PM cell lines using a CCK-8 kit. **(G-H)** Wound-healing assay. OVCAR3 and MR182 cells were plated in a six-well dish. Then linear wounds were created by scraping confluent cell monolayers after the cells were treated with NFIL3 over-expression for 24h. Magnification, ×40. Legends for respective distance are shown to the right of the graph. **(I-J)** Migration assays were performed using HO 8910 and HO 8910PM cells with sgNFIL3 plasmids by transwell assay. Magnification, ×100. Legends for number of migration cells are shown to the bottom of the graph. **(K-N)** Migration of Jurkat cells into the lower chamber was measured after NFIL3 overexpression or knowdown in A2780 and SKOV3. Magnification, ×100. **Compared into NC group, P<0.01. Legends for number of migration cells are shown to the bottom of the graph. *p<0.05, **p<0.01, ***p<0.001. Experiment was repeated 3 times (n = 3).

To assess the potential impact of NFIL3 on the biological processes of ovarian cancer (OC) cells, we conducted an analysis of NFIL3 gene expression in various human ovarian cancer cell lines using western blot analysis (Figure 7A). For the NFIL3 overexpression experiment, we selected the OVCAR3 and MR182 cell lines. In these cell lines, we introduced NFIL3 plasmids to achieve overexpression, which we confirmed through both qRT-PCR and western blot analysis (Figure 7B).

Subsequently, we evaluated the effects of sgNFIL3 knockdown on mRNA expression in HO 8910PM cells (Figure 7C) and protein expression in A2780 cells (Figure 7D). Overexpression of NFIL3 and sgNFIL3 knockdown had substantial effects, resulting in significant changes in both mRNA and protein levels. CCK8 assays provided clear evidence that NFIL3 overexpression significantly inhibited the proliferation of ovarian cancer (OC) cells, while the use of sgRNA targeting NFIL3 promoted OC cell proliferation (Figure 7E-F). Scratch wound healing assays demonstrated that NFIL3 overexpression suppressed cell migration, while NFIL3 knockdown enhanced the migratory ability of ovarian cancer cells (Figure 7G-H). Similarly, transwell assays confirmed that NFIL3 knockdown significantly promoted the migration ability of ovarian cancer cells (Figure 7I-J). As observed in Figure 5A, there was a strong relationship between NFIL3 expression and T cell infiltration. To assess the role of NFIL3 in promoting immune cell infiltration, we conducted transwell migration assays using Jurkat T cells. The results indicated that NFIL3 overexpression increased the capacity of ovarian cancer cells to attract Jurkat T cells (Figure 7K-L), whereas NFIL3 knockdown had the opposite effect, inhibiting the ability of cancer cells to attract Jurkat T cells (Figure 7M-N).

### NFIL3 promotes p53 signaling pathway in ovarian cancer

**Figure 8:**
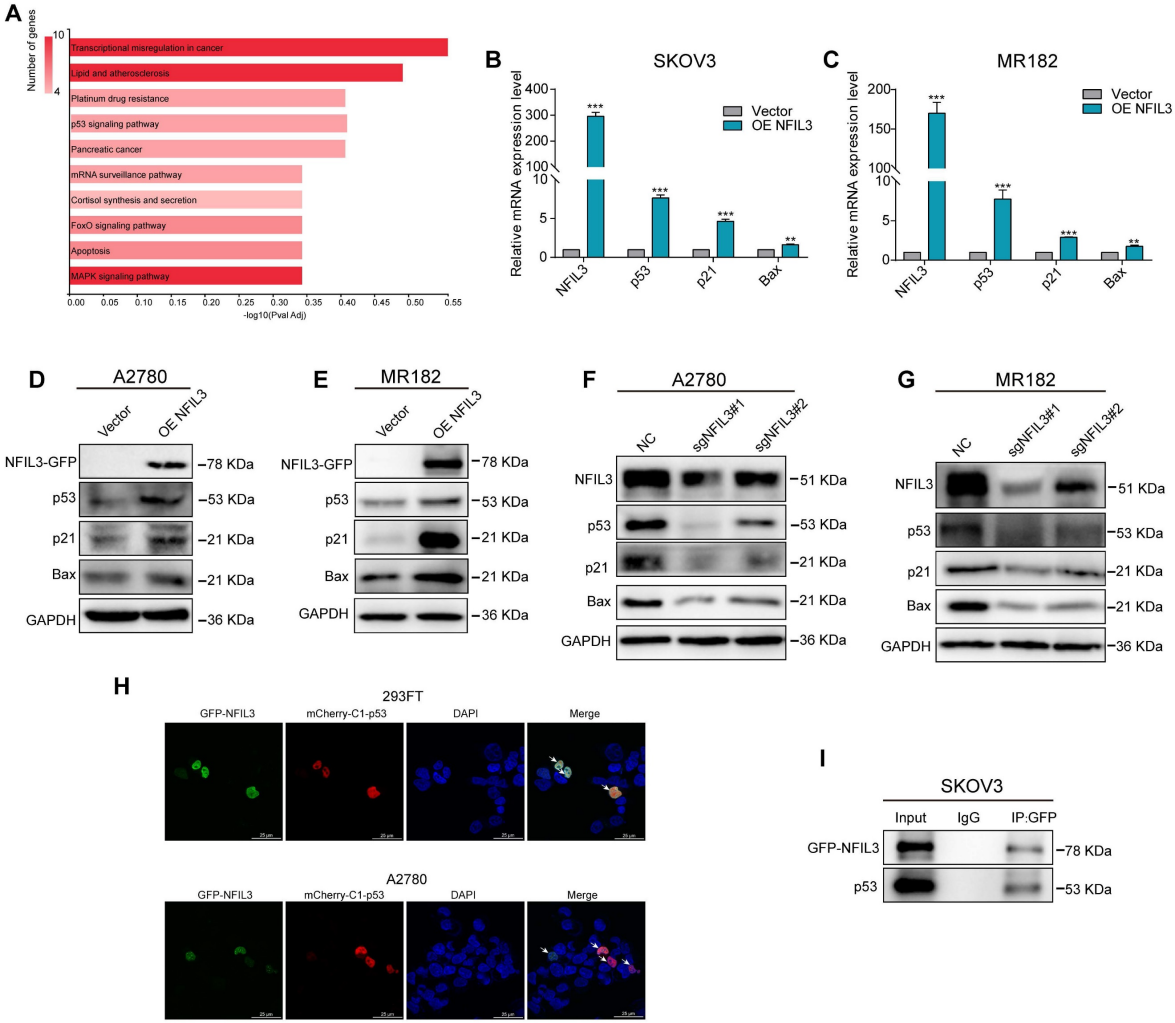
** NFIL3 involves in regulating p53 signaling pathway. (A)** KEGG enrichment analysis of the intersection of NFIL3 targets and the differential gene expression between normal ovary and ovarian cancer tissue. **(B-C)** qRT-PCR analysis of P53, P21 and Bax expression in SKOV3 and MR182 cells. GAPDH serves as an internal control. **(D-E)** The protein levels of NFIL3, p53, p21 and BAX were monitored by western blot after overexpression of NFIL3 in A2780 and MR182 cells. **(F-G)** The protein levels of NFIL3, p53, P21 and BAX were examined by WB after knockdown of NFIL3 in A2780 and MR182 cells. GAPDH serves as an internal control. **(H)** P53 was colocalized with NFIL3 in confocal analysis in A2780 and 293FT, as indicated by white arrow. Scale bar is 25 μm. **(I)** The interaction of p53 and NFIL3 by Co-IP in SKOV3. *p<0.05, **p<0.01, ***p<0.001. Experiment was repeated 3 times (n = 3).

To investigate the mechanistic involvement of NFIL3 in ovarian cancer regulation, we conducted KEGG enrichment analysis on the overlap of potential targets of NFIL3 and genes exhibiting differential expression between normal ovarian tissue and ovarian cancer tissue (as illustrated in Figure 8A). The results highlighted NFIL3's participation in several cancer-related pathways, including transcriptional misregulation in cancer, the p53 signaling pathway, pancreatic cancer, the FoxO signaling pathway, apoptosis, and the MAPK signaling pathway, among others.

Of particular note, the p53 signaling pathway is a well-established classic tumor suppressor pathway. We examined the expression levels of both mRNA and proteins associated with this pathway, such as p53, p21, and Bax. Our findings revealed that overexpression of NFIL3 led to an increase in the mRNA and protein levels of p53, p21, and Bax (as shown in Figure 8B-E). Conversely, knockdown of NFIL3 in A2780 and MR182 cells resulted in a decrease in the protein expression of NFIL3, p53, p21 and Bax (Figure 8F-G).

To ascertain whether a direct interaction exists between p53 and NFIL3, we conducted immunofluorescence confocal microscopy and Co-Immunoprecipitation (Co-IP) experiments. Immunofluorescence confocal experiments, red and green represent P53 and NFIL3 respectively, blue represents nuclei and yellow represents co-localization in A2780 and 293FT cell lines (Figure 8H). SKOV3 cells transiently transfected with NFIL3-GFP or pmCherry-C1-P53 were subjected to GFP pull-down (Figure 8I). Both immunofluorescence confocal microscopy and Co-IP assays provided compelling evidence of a direct physical connection between p53 and NFIL3.

## Discussion

NFIL3, belonging to the basic leucine zipper (bZIP) transcription factor superfamily, plays a multifaceted role in various biological processes and has been associated with several diseases and cellular functions. It has been implicated in neuronal regeneration 23,24, NK cell development 25,26, NF-kappa B (NF-κB) signaling 15,27, circadian clock regulation 28,29, cellular survival 7, heart development and aging 30, osteoblast signal transduction 31, immune regulation 32-34, and cancer development 7,8,35-37.

In the context of cancer, NFIL3 has shown diverse roles. For example, it has been linked to both favorable and unfavorable prognosis in different cancer types, such as breast cancer and colon cancer. Mechanistically, NFIL3 has been found to block FOXO1 recruitment to certain tumor suppression-related genes and prevent the recruitment of Proline Acid Rich (PAR) transcription factors to pro-apoptotic genes in colon cancer.

A comprehensive study was conducted to investigate NFIL3 expression in human cancers and corresponding normal tissues, revealing that NFIL3 has lower expression levels in cancers compared to normal tissues, suggesting a potential tumor-suppressive role for NFIL3. Furthermore, a protein-protein interaction (PPI) network analysis identified several genes associated with NFIL3, some of which play crucial roles in cancer development.

The study also examined the correlation between NFIL3 expression and the prognosis of various cancer types. High NFIL3 expression was associated with better prognosis in breast cancer and ovarian cancer but worse prognosis in colon cancer, consistent with previous studies. Additionally, NFIL3 has been implicated in the progression of triple negative breast cancer (TNBC) by activating NF-κB signaling.

Notably, the study explored the relationship between NFIL3 gene expression and non-synonymous single nucleotide variations (SNVs) in multiple cancers, shedding light on potential mutation patterns that could inform cancer diagnosis and treatment. Additionally, the analysis of immune cell infiltration suggested a significant association between NFIL3 expression and immune cell presence, along with immune checkpoint gene correlations.

In ovarian cancer, NFIL3 was found to exert significant effects on cell proliferation, migration, and immune cell infiltration. NFIL3 overexpression inhibited cell proliferation and migration while promoting immune cell infiltration. Conversely, NFIL3 knockdown had the opposite effects.

Further investigation into the role of NFIL3 in ovarian cancer revealed a positive correlation between NFIL3 and p53 target gene expression. NFIL3 overexpression increased the expression of p53, p21, and BAX, while NFIL3 knockdown reduced their expression. Co-immunoprecipitation (Co-IP) and immunofluorescence analyses indicated a physical association between p53 and NFIL3, suggesting a potential tumor suppressor role for NFIL3 via the p53 signaling pathway.

In conclusion, this comprehensive study highlights the complex and multifaceted role of NFIL3 in cancer biology, emphasizing its potential as both a diagnostic marker and a therapeutic target in various cancer types.

## Conclusions

NFIL3 may be a novel biomarker with potential prognostic and immunotherapy roles in pan-cancer. Especially, NFIL3 targeted therapy may be a suitable candidate for treatment of OC.

## Supplementary Material

Supplementary tables.

## Abbreviations

Abbreviations of all cancers are given in Supplementary Table S1.
