# Supplementary material for: A pan-cancer characterization of immune-related NFIL3 identifies potential predictive biomarker
Source: J Cancer. 2024 Jan 12;15(5):1271–86. doi: 10.7150/jca.88765 (PMC10861811; doi:10.7150/jca.88765)
Supplement: Supplementary file 1 — Supplementary tables. [file jcav15p1271s1.pdf]

Supplementary Table S1

**Abbreviations**	**Cancer Types**
ACC	Adrenocortical carcinoma
ALL	Acute lymphocytic leukemia
BLCA	Bladder Urothelial Carcinoma
BRCA	Breast invasive carcinoma
CESC	Cervical squamous cell carcinoma and endocervical adenocarcinoma
CHOL	Cholangiocarcinoma
COAD	Colon adenocarcinoma
COADREAD	Colon adenocarcinoma/Rectum adenocarcinoma Esophageal
DLBC	Lymphoid Neoplasm Diffuse Large B-cell Lymphoma
ESCA	Esophageal carcinoma
FPPP	FFPE Pilot Phase II
GBM	Glioblastoma multiforme
GBMLGG	Glioma
HNSC	Head and Neck squamous cell carcinoma
KICH	Kidney Chromophobe
KIPAN	Pan-kidney cohort (KICH+KIRC+KIRP)
KIRC	Kidney renal clear cell carcinoma
KIRP	Kidney renal papillary cell carcinoma
LAML	Acute Myeloid Leukemia
LGG	Brain Lower Grade Glioma
LIHC	Liver hepatocellular carcinoma
LUAD	Lung adenocarcinoma
LUSC	Lung squamous cell carcinoma
MESO	Mesothelioma
OV	Ovarian serous cystadenocarcinoma
OC	Ovarian Cancer
PAAD	Pancreatic adenocarcinoma
PCPG	Pheochromocytoma and Paraganglioma
PRAD	Prostate adenocarcinoma
READ	Rectum adenocarcinoma
SARC	Sarcoma
SKCM	Skin Cutaneous Melanoma
STAD	Stomach adenocarcinoma
STES	Stomach and Esophageal carcinoma
TGCT	Testicular Germ Cell Tumors
THCA	Thyroid carcinoma
THYM	Thymoma
UCEC	Uterine Corpus Endometrial Carcinoma
UCS	Uterine Carcinosarcoma
UVM	Uveal Melanoma
WT	Warthin tumor

Table S2

Primers sequences for sgRNA clone

Primers	Sequence
NFIL3-sg1-F	CACCGAGGGAGCCAAGAGATGACCG
NFIL3-sg1-R	AAACCGGTCATCTCTTGGCTCCCTC
NFIL3-sg2-F	CACCGGTTGTTGAAATGAAAGACAG
NFIL3-sg2-R	AAACCTGTCTTTCATTTCAACAACC
NFIL3-sg3-F	CACCGTGGCATCAAAAAGAACTGAG
NFIL3-sg3-R	AAACCTCAGTTCTTTTTGATGCCAC
NFIL3-sg4-F	CACCGACAACTACTTGACACCATCG
NFIL3-sg4-R	AAACCGATGGTGTCAAGTAGTTGTC
